# Status and influencing factors of medication literacy among Chinese caregivers of discharged children with Kawasaki disease

**DOI:** 10.3389/fpubh.2022.960913

**Published:** 2022-10-17

**Authors:** Yingzi Zhang, Xiuqiong Wang, Jianghui Cai, Yanfeng Yang, Yiling Liu, Yeling Liao, Yanhong Zhou, Baoqin He, Wen Wen, Qian Zhuang, Yonghong Lin

**Affiliations:** ^1^Department of Pediatric Cardiology, Chengdu Women's and Children's Central Hospital, School of Medicine, University of Electronic Science and Technology of China, Chengdu, China; ^2^Department of Pharmacy, Chengdu Women's and Children's Central Hospital, School of Medicine, University of Electronic Science and Technology of China, Chengdu, China; ^3^Department of Obstetrics and Gynecology, Chengdu Women's and Children's Central Hospital, School of Medicine, University of Electronic Science and Technology of China, Chengdu, China

**Keywords:** Kawasaki disease, caregivers, medication literacy, Medication Literacy Assessment, influencing factors

## Abstract

**Background:**

The information on medication literacy among Chinese caregivers of discharged children with Kawasaki disease (KD) is unknown. We aimed to investigate the status of medication literacy among caregivers of discharged children with KD and evaluate the influencing factors of medication literacy.

**Methods:**

From March 2020 to February 2021, 106 caregivers with a KD child were recruited for the present study. We collected the sociodemographic characteristics of the KD caregivers using structured interviews. The medication literacy of the KD caregivers was assessed by the Chinese version of Medication Literacy Assessment. KD patients' demographic and clinical data were obtained from the medical records. The multiple logistic regression was performed to identify factors associated with medication literacy.

**Results:**

(1) The average medication literacy score was 4.91 ± 1.51. (2) Most of the Chinese KD caregivers had insufficient medication literacy (≤ 5 scores), and only 39.2% of the caregivers had adequate medication literacy (>5 scores). (3) The multiple logistic regression shows that education level, monthly income, and duration of hospitalization are the independent influencing factors on the medication literacy of KD caregivers.

**Conclusion:**

There is preliminary evidence that medication literacy among KD caregivers is low and needs improvement. A higher level of education, higher income, and longer duration of hospitalization were influencing factors of adequate medication literacy.

## Introduction

Medication literacy (ML) was first mentioned in 2005 in a government document to provide medication information to individuals with low health literacy (1). The international perspective on the definition of medication literacy is the degree to which individuals can obtain, comprehend, communicate, calculate, and process patient-specific information about their medication to make informed medication and health decisions to safely and effectively use their medications regardless of the mode by which the content is delivered (e.g., written, oral, and visual) (2). ML can be a critical predictor of rational medication use and significantly impacts medication safety in clinical practice (3, 4). Patients with a higher level of ML usually had better medication compliance (5, 6). On the contrary, patients with limited ML may have problems understanding medication information and undergo more frequent re-hospitalization, emergency department visits, and serious adverse drug events (5, 7–9). In addition, previous studies have found that most medication-related adverse events would be preventable with better medication literacy (10, 11). Thus, ML determines how well patients can manage their medication regimens correctly and plays an important role in reducing and avoiding adverse drug events (12, 13).

Kawasaki disease (KD) is an acute, self-limited febrile illness of unknown cause that predominantly affects children < 5 years of age (14). KD is now recognized as a leading cause of acquired heart disease in children in developed countries. KD has been reported in more than 60 countries since first described in Japan (15–25). Patients with KD require long-term medication after discharge. It is critical for patients with KD to use medicine regularly and correctly over the long term to reduce the occurrence of coronary artery lesions.

There is an urgent need to investigate the status of medication literacy among caregivers of discharged children with KD, as most KD kids cannot take care of themselves and use medicine appropriately. However, the medication literacy among Chinese caregivers of discharged children with KD remains unknown. There is an urgent need in clinical practice to appraise available evidence and evaluate where gaps exist. A survey showed that about 70% of preventable adverse drug events in pediatric outpatients were due to parents' lack of knowledge about medication administration (26). Considering that China is one of the most populous countries and the increasing incidence of KD (22, 27, 28), it is imperative to understand the medication literacy status among Chinese caregivers of discharged children with KD. To the best of our knowledge, no study has explored medication literacy among Chinese caregivers of discharged children with KD. Therefore, we aimed to preliminarily investigate medication literacy among Chinese KD caregivers and examine the influencing factors of medication literacy.

## Methods

### Ethics and informed consent

This study received approval from the Research Ethics Committee of Chengdu Women's and Children's Central Hospital (NO. 202013). All participants gave their written informed consent for inclusion before the study.

### Study setting

Caregivers of discharged KD patients were recruited from the pediatric cardiology department of Chengdu Women's and Children's Central Hospital from March 2020 to February 2021. The caregiver was defined as the person responsible for attending to the needs of a child with KD. Detailed information on the proportion of the relationship between KD patients and caregivers can be seen in Appendix S1.

### Participants

Discharged children with KD and caregivers were eligible if they met the following inclusion criteria:

- Patients < 18 years old; AND
- The diagnosis of complete or incomplete KD was established according to the American Heart Association guideline in 2017 (14); AND
- Patients of initial onset of KD; AND
- KD patients without any major medical conditions; AND
- Currently taking at least one medication; AND
- Informed consent was obtained from all participants; AND
- Caregivers have normal cognitive function and competent communication ability.

Exclusion criteria were as follows:

- The patient was previously diagnosed with KD and had a recurrence; OR
- Caregivers were diagnosed with any psychiatric disorders; OR
- Caregivers had medical work experience; OR
- Unwilling to participate in the survey.

All KD kids received the standard therapy, which included high doses of IVIG (2 g/kg) as a single infusion and aspirin (30–50 mg/kg/d during the acute phase of illness) immediately after the diagnosis. The dose of aspirin was lowered to 3-5 mg/kg/d after the child had been afebrile for 48–72 h. The 2nd IVIG of the same dosage was administrated for KD patients with initial IVIG-resistant. If fever persists 36 h after the 2nd IVIG infusion, intravenous methylprednisolone (30 mg/kg/dose) was performed for 3 consecutive days. No other therapies, including infliximab, plasma exchange, and cytotoxic agents, were used in the treatment protocol. Initial IVIG resistance was defined as recurrent or persistent fever for at least 36 h but not longer than 7 days after initial IVIG treatment (14). CAL was defined as a coronary artery internal diameter with a z score of ≥2.5 in at least one of the following coronary arteries: left, right anterior descending, and left-main (29).

### Data collection tools and survey procedures

#### Sociodemographic characteristics of KD caregivers

On the day of discharge, KD patients' demographic and clinical data, such as age, male/female, and length of hospitalization, were retrieved from the electronic medical records. The attending doctor provided instructions for the drugs to the KD caregivers, including the names, therapeutic effects, dosage, frequency of use, and main side effects at discharge. There were no teaching sessions related to medicine for KD caregivers during the hospital stay.

Eligible KD caregivers were invited to participate in the study and provided with information on the study objectives, study content, and investigation procedures. The survey was carried out through face-to-face interviews using the paper-and-pencil method. A self-developed and structured questionnaire designed by a researcher (BH) was used to obtain information on the sociodemographic variable of the KD caregivers. The questionnaires were required to be filled out on the spot. Caregivers of discharged children with KD completed questionnaires anonymously after giving signed informed consent. For illiterate participants, the interviewers read the question items word by word exactly, and participants' responses were recorded on the questionnaires. The questionnaires were collected immediately after completion, checked for missing information, and followed up with the participants.

#### The Chinese version of the Medication Literacy Assessment

Due to the COVID-19 pandemic, stringent rules for pandemic protection measures were diligently applied. We assess the medication literacy of KD caregivers by telephone. Twenty days after discharge, the interviewers made telephone calls to the KD caregivers to assess their medication literacy based on an outlined structured interview. All patients had at least one follow-up in the outpatient department from hospital discharge before ML assessment. Medication literacy was measured by the Chinese version of the Medication Literacy Assessment for Discharged Patients (MedLitRxSE-Chinese). Maniaci and colleagues first proposed the Medication Literacy Assessment in English (MedLitRxSE-English) (30). The MedLitRxSE-English was used to measure the ability of patients to read, comprehend, calculate, and cope with medication-related problems in the medical information environment to assess their level of medication literacy (31). The MedLitRxSE-Chinese is a self-administered scale. It was introduced by Zheng et al. (32) according to the principles of Brislin and culture adjustment (33). The questionnaire assessed the patient's ability to understand, calculate, and process drug information.

The MedLitRxSE-Chinese includes nine items with a dichotomy scoring system (1 for correct answers and 0 for errors). Item 7 has only a “Yes” or “No” answer, and item 9 has specific names. Therefore, items 7 and 9 do not contribute to the total score. As a result, the maximum scale score is 7. The total score ranges from 0 to 7, and a higher score reflects a higher level of medication literacy. The scores were divided into three groups: adequate literacy (≧6), marginal literacy (4-5), and inadequate literacy (< 4). The MedLitRxSE-Chinese has good reliability and validity in Chinese populations (Cronbach's α coefficient = 0.850, content validity index of the questionnaire = 0.812, and retesting reliability coefficient = 0.94) (32, 34). Specifically, in item 3, the original question was: “Did you know the generic names of the medicines that your kids are taking”. Considering the name of drugs may be difficult to know by all KD caregivers as it is medical jargon. The brand names of medicines seem to be easier to bear in mind. So we changed the question to: “Did you know the generic or brand names of the medicines your kids are taking”.

Before conducting the Medication Literacy Assessment, the researchers obtained oral informed consent. Each KD caregiver was required to answer the MedLitRxSE-Chinese. The investigators checked the nine scales through an electronic medical record system to determine whether the answer was correct. The flow chart of the survey process can be seen in Appendix S2.

### Data analysis

Continuous variables were directly expressed as a range. Categorical variables were expressed by presenting the frequency and proportion in each category. Demographic information was analyzed by descriptive statistical analysis. We used *t*-tests, analysis of variance, and the Kruskal–Wallis H test for the univariate analysis. Logistic regression analysis was used to analyze the independent influencing factors of medication literacy. All *P*-values were from 2-sided tests, and the results were considered statistically significant at *P* < 0.05. Statistical analysis was performed with SPSS (version 22.0, Chicago, U.S.).

## Results

### General characteristics of caregivers

From March 2020 to February 2021, 106 caregivers of discharged children with KD who met the inclusion criteria were included in the present study. Nine were further excluded in the data cleaning stage because of missing variables. A total of 97 completed questionnaires were received, with a valid response rate of 91.5%. There were no significant differences in gender of KD caregivers, education level, income, employment status and follow-up intervals. Meanwhile, there were significant differences in the age of KD caregivers, type of KD patients (complete VS. incomplete), the complication of KD patients (CAL VS. non-CAL), and duration of hospitalization (*P* < 0.05). The general characteristics of KD caregivers and patients are presented in Table 1.

**Table 1:** General characteristic of KD caregivers and KD patients.

**Characteristics**	**Items**	**No. of participants (*N* = 97)**	**Percentage (%)**
			
**Age of KD caregivers (years)**			
	≤ 35	65	67.01
	>35	32	32.99
**Gender of KD caregivers**			
	Male	50	51.55
	Female	47	48.45
**Education level**			
	Middle school and below	20	20.62
	High school	30	30.93
	College	29	29.90
	Master/Doctoral	18	18.55
**Income (monthly)**			
	< 2,000 Yuan	30	30.93
	2,001–5,000 Yuan	23	23.71
	5,001–9,000 Yuan	23	23.71
	>9,000 Yuan	21	21.65
**Employment status**			
	Full-time/Part-time	52	53.61
	Unemployed	45	46.39
**Follow-up intervals (days)**			
	≤ 7 days	54	55.67
	>7 days	43	44.33
**Duration of hospitalization (days)**			
	≤ 5 days	37	38.14
	>5 days	60	61.86
**Age of KD patients (years)**			
	< 5	40	41.24
	≥5	57	58.76
**Number of medicines the KD patients currently taken**			
	1	1	1.03
	2-3	83	85.57
	≥4	13	13.40
**Type of KD patients (n)**			
	Complete KD	68	70.10
	Incomplete KD	29	29.90
**Complication of KD patients**			
	CAL	19	19.59
	Non-CAL	78	80.41

CAL, Coronary artery lesions; Non-CAL, Non-coronary artery lesions.

### Medication literacy of caregivers of discharged children with KD

In brief, the mean score of medication literacy was 4.91 (1.51). Caregivers who obtained a score ≧ of 6 were 39.2% (38) and considered adequate medication literacy. Those who obtained a score of 4-5 were 39.2% (38) and were considered marginal medication literacy, whereas 21.6% (21) obtained a score < 4 and were considered inadequate medication literacy. Although all caregivers knew their kids had to take medicine after discharge, only 86.6% knew how many kinds of drugs their kid should take daily, and 76.3% could name the medications their kid was taking. 78.4% knew the frequency of taking medications every day, only 38.1% knew the effects, and 32.9% had been informed of the side effects of drugs. Furthermore, only 19.6% knew the side effects of each kind of medicine their kid was taking (Table 2).

**Table 2:** Medication literacy for caregivers of discharged children with KD.

**Items**	**Number of** **correct answers** **no. (%) (*n =* 97)**
1. Did your kids take medicines after discharge from hospital?	97 (100%)
2. How many kinds of medicines did your kids need to take every day?	84 (86.6%)
3. Did you know the generic or brand names of the medicines that your kids are taking?	74 (76.3%)
4. Did your know the dosage of each kinds of medicine that your kids are taking?	92 (94.8%)
5. Did you know how many times a day for each kind of medicine?	76 (78.4%)
6. Do you know the purpose (effect) of each kind of drug?	37 (38.1%)
7. Have you ever been warned of side effects of the medicines that your kids are taking?	32 (32.9%)
8. Did you know the common side effects of each kinds of medicine your child is taking?	19 (19.6%)
9. Did you know whom you should consult with in case of questions related to the medicines you are taking?	
A. Physician	6 (6.2%)
B. Pharmacist	63 (64.9%)
C. Nurse	23 (23.7%)
D. I have no idea	3 (3.1%)
E. Others	2 (2.1%)

### Influencing factors of medication literacy

Table 3 shows the results of the univariate analysis. Five factors were significantly associated with medication literacy, with higher scores observed in highly educated, a higher income, unemployed, shorter follow-up intervals, and longer duration of hospitalization.

**Table 3:** Univariate analysis of determinants of medication literacy for KD caregivers.

**Variables**	**No**.	**The score of ML**			**t/H/χ^2^**	***P***
		**0–3**	**4–5**	**6–7**		
**Age of KD caregivers (years)**					4.619	0.099
≤ 35	65	10	28	27		
>35	32	11	10	11		
**Gender of KD caregivers**					0.317	0.853
Male	46	11	17	18		
Female	51	10	21	20		
**Education level**					52.555	< 0.001
Middle school and below	20	16	3	1		
High school	30	2	16	12		
College	29	2	12	15		
Master/Doctoral	18	1	7	10		
**Income (monthly)**					46.240	< 0.001
< 2,000 Yuan	30	15	8	7		
2,001–5,000 Yuan	23	6	11	6		
5,001–9,000 Yuan	23	0	16	7		
>9,000 Yuan	21	0	3	18		
**Employment status**					12.646	0.002
Full-time/Part-time	52	13	27	12		
Unemployed	45	8	11	26		
**Follow-up intervals**					−3.792	< 0.001
≤ 7 days	54	4	22	28		
>7 days	43	17	16	10		
**Duration of hospitalization**					18.161	< 0.001
≤ 5 days	37	15	16	6		
>5 days	60	6	22	32		
**Age of KD patients (years)**					0.083	0.959
< 5	40	9	15	16		
≥5	57	12	23	22		
**Number of medicines the KD patients currently taken**					4.957	0.292
1	1	/	/	1		
2–3	83	17	31	35		
≥4	13	4	7	2		
**Type of KD patients**					1.592	0.451
Complete KD	68	15	24	29		
Incomplete KD	29	6	14	9		
**Complication of KD patients**					4.074	0.13
CAL at the acute phase	19	1	10	8		
Non-CAL at the acute phase	78	20	28	30		

t, two-sample t-test; H, Kruskal–Wallis test; CAL, Coronary artery lesions; Non-CAL, Non-coronary artery lesions.

The multiple logistic regression shows that education level, monthly income, and duration of hospitalization are the independent influencing factors on medication literacy of KD caregivers. Medication literacy scores increased with the educational level, monthly income, and length of hospitalization (Table 4).

**Table 4:** Multiple logistic regression of influencing factors among KD caregivers.

**Determinants**	***B***	**S.E**.	**Wald**	***P***	**OR**	**95%C.I. of OR**	
						**Lower**	**Upper**
Education level	1.461	0.544	7.21	< 0.001	4.311	1.484	12.524
Income (monthly)	2.249	0.622	13.071	< 0.001	9.474	2.8	32.058
Employment status (Employed)	−2.563	1.333	3.694	0.055	0.077	0.006	1.052
Follow-up intervals	−0.237	0.156	2.326	0.127	0.789	0.582	1.07
Duration of hospitalization	0.399	0.169	5.545	0.019	1.49	1.069	2.076

B, Partial regression coefficient; SE, Standard error; CI, Confidence intervals.

## Discussion

Our study described the status of medication literacy among Chinese caregivers of discharged children with KD and explored possible influencing factors of medication literacy. Our findings showed that most of the Chinese KD caregivers had insufficient medication literacy (≤ 5 scores), and only 39.2% of the caregivers had adequate medication literacy (>5 scores). Meanwhile, our study also found that educational level, monthly income, and length of hospitalization were independently associated with medication literacy.

To properly use their medications, patients must read the related medical information, including medication labels and instructions, and take the accurate dose. Our study is of obvious importance, considering that KD predominantly affects children < 5 years of age. Most KD patients lack medication adherence and do not know how to self-medication properly. Thus, knowing the status and exploring the influencing factors of medication literacy among KD is essential.

Our study showed that medication literacy among KD caregivers needs improvement as the overall medication literacy scores are low, consistent with previous reports of Chinese populations (10, 35). Thus, it is critical to improve the medication literacy of the KD caregivers as inappropriate medication use was identified to be significantly associated with low medication literacy (6, 8, 10).

Several factors were associated with higher medication literacy. Our findings are consistent with the results of other studies that showed higher education levels and higher income were significantly associated with a higher medication literacy score (13, 31, 36, 37). Highly educated obtain higher medication literacy scores indicating that general knowledge may contribute to understanding medication information. The higher the educational level of caregivers, the stronger their ability to obtain medication information, the wider the channels for obtaining information, and the stronger the ability to make correct medication decisions (38). It was worth noting that most caregivers in this population had a low education level and limited knowledge of medication regimes, indicating that they are prone to trusting physicians about their treatment. Higher monthly income is another influencing factor, suggesting that caregivers with higher incomes might have more access to medication knowledge and were more likely to pay more attention to promoting health levels. Therefore, caregivers who are less educated and earned less should be targeted for medication literacy improvement.

Duration of hospitalization was another independent factor affecting medication literacy. We found that those with a longer duration of hospitalization were more likely to have a higher level of medication literacy, which is consistent with an early study (39). It indicated that medical staff should focus on caregivers with shorter duration. A possible explanation was the longer the patients stayed in the hospital, the more health information their caregivers received from doctors and other nurses. Meanwhile, these caregivers may pay more attention to their kids' conditions while in the hospital, thus improving their medication literacy.

Based on the results of this study, we provide several suggestions to improve medication literacy among KD caregivers. First, effective communication between caregivers and health care providers is needed. Second, high-quality and comprehendible education materials (e.g., booklets, online medical information) should be designed as caregivers sometimes cannot remember detailed medication information and guidance verbally communicated by physicians. Third, provide a training education to improve the nursing knowledge and level of Kawasaki disease among medical staff. Besides, regular follow-ups are important methods to gain medication knowledge and improve medication literacy.

## Limitations

This study has potential limitations. First, our study had a small sample size. Second, all the caregivers came from Sichuan, southwest of China, which limited the generalization of the findings. Future studies should focus on caregivers from other districts and larger sample sizes. Third, we didn't collect data on where the caregivers learned information about the medication or their preferred way of receiving it. This could help medical care institutions improve their future services and the medical literacy level of their patients' caregivers. Four, the telephone call-based assessment may introduce bias.

## Conclusion

The overall level of medication literacy among KD caregivers is low and needs to be improved. A higher level of education, higher income, and longer duration of hospitalization were influencing factors of adequate medication literacy. However, further multiple-center research with bigger sample size is needed to find effective measures to improve medication literacy among caregivers.

## Data availability statement

The raw data supporting the conclusions of this article will be made available by the authors, without undue reservation.

## Ethics statement

The studies involving human participants were reviewed and approved by the Research Ethics Committee of Chengdu Women's and Children's Central Hospital. Written informed consent to participate in this study was provided by the participants' legal guardian/next of kin.

## Author contributions

YY had full access to all of the data in the study and takes responsibility for the integrity of the data. YZha, XW, and JC drafted the manuscript. JC contributed to the design of the search strategy. YLia, YZho, WW, and QZ collected the data. BH designed the self-developed and structured questionnaire. YLia and YLin did the statistical analysis. All authors read, provided feedback, and approved the final version.

## Conflict of interest

The authors declare that the research was conducted in the absence of any commercial or financial relationships that could be construed as a potential conflict of interest.

## Publisher's note

All claims expressed in this article are solely those of the authors and do not necessarily represent those of their affiliated organizations, or those of the publisher, the editors and the reviewers. Any product that may be evaluated in this article, or claim that may be made by its manufacturer, is not guaranteed or endorsed by the publisher.

## References

[1] Raynor DK. Medication literacy is a 2-way street. Mayo Clin Proc. (2008) 83:520–2. 10.4065/83.5.520 18452678

[2] Pouliot A Vaillancourt R Stacey D Suter P. Defining and identifying concepts of medication literacy: an international perspective. Res Social Adm Pharm. (2018) 14:797–804. 10.1016/j.sapharm.2017.11.005 29191647

[3] Cordina M Hämeen-Anttila K Lauri J Tabone S Enlund H. Health and medication literacy and the desire to participate in pharmacotherapy decision making - comparison of two countries. Res Social Adm Pharm. (2018) 14:817–23. 10.1016/j.sapharm.2018.06.009 29934278

[4] Raynor DK. Addressing medication literacy: a pharmacy practice priority. Int J Pharm Pract. (2009) 17:257–9. 10.1211/ijpp.17.05.0001 20214266

[5] Zhong Z Zheng F Guo Y Luo A. Medication literacy in a cohort of Chinese patients discharged with Acute Coronary Syndrome. Int J Environ Res Public Health. (2016) 13:720. 10.3390/ijerph13070720 27428990 PMC4962261

[6] Lee CH Chang FC Hsu SD Chi HY Huang LJ Yeh MK. Inappropriate self-medication among adolescents and its association with lower medication literacy and substance use. PLoS ONE. (2017) 12:e0189199. 10.1371/journal.pone.0189199 29240799 PMC5730183

[7] Zhang J Gilmour S Liu Y Ota E. Effect of health literacy on quality of life among patients with chronic heart failure in China. Qual Life Res. (2020) 29:453–61. 10.1007/s11136-019-02332-4 31628646

[8] Kripalani S Henderson LE Jacobson TA Vaccarino V. Medication use among inner-city patients after hospital discharge: patient-reported barriers and solutions. Mayo Clin Proc. (2008) 83:529–35. 10.4065/83.5.529 18452681

[9] Neiva Pantuzza LL Nascimento ED Crepalde-Ribeiro K Botelho SF Parreiras Martins MA Camila de Souza Groia Veloso R. Medication literacy: a conceptual model. Res Social Adm Pharm. (2022) 18:2675–82. 10.1016/j.sapharm.2021.06.003 34134939

[10] Zheng F Ding S Luo A Zhong Z Duan Y Shen Z. Medication literacy status of outpatients in ambulatory care settings in Changsha, China. J Int Med Res. (2017) 45:303–9. 10.1177/0300060516676726 28222647 PMC5536586

[11] Pouliot A Vaillancourt R. Medication literacy: why pharmacists should pay attention. Can J Hosp Pharm. (2016) 69:335–6. 10.4212/cjhp.v69i4.1576 27621498 PMC5008434

[12] Mosher HJ Lund BC Kripalani S Kaboli PJ. Association of health literacy with medication knowledge, adherence, and adverse drug events among elderly veterans. J Health Commun. (2012) 17 Suppl 3:241–51. 10.1080/10810730.2012.712611 23030573

[13] Shen Z Shi S Ding S Zhong Z. Mediating effect of self-efficacy on the relationship between medication literacy and medication adherence among patients with hypertension. Front Pharmacol. (2020) 11:569092. 10.3389/fphar.2020.569092 33364943 PMC7750474

[14] McCrindle BW Rowley AH Newburger JW Burns JC Bolger AF Gewitz M. Diagnosis, treatment, and long-term management of Kawasaki disease: a scientific statement for health professionals from the American Heart Association. Circulation. (2017) 135:e927–99. 10.1161/CIR.0000000000000484 28356445

[15] Al-Ammouri I Al-Wahsh S Khuri-Bulos N. Kawasaki disease in Jordan: demographics, presentation, and outcome. Cardiol Young. (2012) 22:390–5. 10.1017/S1047951111001818 22067065

[16] Alexopoulos A Vekiou A Lycopoulou L Tavena A Lagona E Kakourou T. Kawasaki disease in Greek children: a retrospective study. J Eur Acad Dermatol Venereol. (2013) 27:580–8. 10.1111/j.1468-3083.2012.04488.x 22360836

[17] Arkachaisri T. Pediatric rheumatology in Southeast Asia: insights from the Singapore experience. Curr Rheumatol Rep. (2011) 13:117–22. 10.1007/s11926-010-0159-1 21181313

[18] Bar-Meir M Haklai Z Dor M. Kawasaki disease in Israel. Pediatr Infect Dis J. (2011) 30:589–92. 10.1097/INF.0b013e31820e3849 21343843

[19] Davaalkham D Nakamura Y Baigalmaa D Davaa G Chimedsuren O Sumberzul N. Kawasaki disease in Mongolia: results from 2 nationwide retrospective surveys, 1996-2008. J Epidemiol. (2011) 21:293–8. 10.2188/jea.JE20100144 21691035 PMC3899422

[20] Huang SK Lin MT Chen HC Huang SC Wu MH. Epidemiology of Kawasaki disease: prevalence from national database and future trends projection by system dynamics modeling. J Pediatr. (2013) 163:126–31.e121. 10.1016/j.jpeds.2012.12.011 23312687

[21] Singh S Aulakh R Bhalla AK Suri D Manojkumar R Narula N. Is Kawasaki disease incidence rising in Chandigarh, North India? Arch Dis Child. (2011) 96:137–40. 10.1136/adc.2010.194001 20923951

[22] Uehara R Belay ED. Epidemiology of Kawasaki disease in Asia, Europe, and the United States. J Epidemiol. (2012) 22:79–85. 10.2188/jea.JE20110131 22307434 PMC3798585

[23] Salo E Griffiths EP Farstad T Schiller B Nakamura Y Yashiro M. Incidence of Kawasaki disease in northern European countries. Pediatr Int. (2012) 54:770–2. 10.1111/j.1442-200X.2012.03692.x 22726311 PMC3467350

[24] Masuda H Ae R Koshimizu TA Matsumura M Kosami K Hayashida K. Epidemiology and risk factors for giant coronary artery aneurysms identified after acute Kawasaki disease. Pediatr Cardiol. (2021) 42:969–77. 10.1007/s00246-021-02571-8 33682062

[25] Matsubara Y Matsubara D Ae R Kosami K Aoyama Y Yashiro M. Cumulative incidence of Kawasaki disease with cardiac sequelae in Japan. Pediatr Int. (2020) 62:444–50. 10.1111/ped.14164 31960532

[26] Kaushal R Goldmann DA Keohane CA Christino M Honour M Hale AS. Adverse drug events in pediatric outpatients. Ambul Pediatr. (2007) 7:383–9. 10.1016/j.ambp.2007.05.005 17870647

[27] Du ZD Zhang T Liang L Meng X Li T Kawasaki T. Epidemiologic picture of Kawasaki disease in Beijing from 1995 through 1999. Pediatr Infect Dis J. (2002) 21:103–7. 10.1097/00006454-200202000-00004 11840075

[28] Singh S Vignesh P Burgner D. The epidemiology of Kawasaki disease: a global update. Arch Dis Child. (2015) 100:1084–8. 10.1136/archdischild-2014-307536 26111818

[29] McCrindle BW Li JS Minich LL Colan SD Atz AM Takahashi M. Coronary artery involvement in children with Kawasaki disease: risk factors from analysis of serial normalized measurements. Circulation. (2007) 116:174–9. 10.1161/CIRCULATIONAHA.107.690875 17576863

[30] Maniaci MJ Heckman MG Dawson NL. Functional health literacy and understanding of medications at discharge. Mayo Clin Proc. (2008) 83:554–8. 10.1016/S0025-6196(11)60728-3 18452685

[31] Qiao L Ding S Zhong Z Liu X Lai L Zheng F. Association between social support and medication literacy in Chinese patients with coronary heart disease. Front Cardiovasc Med. (2021) 8:705783. 10.3389/fcvm.2021.705783 34901201 PMC8655157

[32] Zheng F Zhong Z Ding S Luo A Liu Z. [Modification and evaluation of assessment of medication literacy]. Zhong Nan Da Xue Xue Bao Yi Xue Ban. (2016) 41:1226–31. 10.11817/j.issn.1672-7347.2016.11.01 27932772

[33] Jones PS Lee JW Phillips LR Zhang XE Jaceldo KB. An adaptation of Brislin's translation model for cross-cultural research. Nurs Res. (2001) 50:300–4. 10.1097/00006199-200109000-00008 11570715

[34] Zheng F Ding S Zhong Z Pan C Xie J Qin C. Investigation on status of discharged patients' medication literacy after coronary artery stent implantation. Chin Nurs Res. (2015) 29:1732–4. 10.3969/j.issn.10096493.2015.14.024

[35] Zheng F Ding S Lai L Liu X Duan Y Shi S. Relationship between medication literacy and medication adherence in inpatients with coronary heart disease in Changsha, China. Front Pharmacol. (2019) 10:1537. 10.3389/fphar.2019.01537 32009954 PMC6974678

[36] Ma G Luo A Shen Z Duan Y Shi S Zhong Z. The status of medication literacy and associated factors of hypertensive patients in China: a cross-sectional study. Intern Emerg Med. (2020) 15:409–19. 10.1007/s11739-019-02187-0 31650433 PMC7165129

[37] Shi S Shen Z Duan Y Ding S Zhong Z. Association between medication literacy and medication adherence among patients with hypertension. Front Pharmacol. (2019) 10:822. 10.3389/fphar.2019.00822 31396088 PMC6664237

[38] Qu J Zhou T Xue M Sun H Shen Y Liu Y. Relationship between medication literacy and frailty in elderly inpatients with coronary heart disease: a cross-sectional study in China. Front Pharmacol. (2021) 12:691983. 10.3389/fphar.2021.691983 34305601 PMC8295746

[39] Zhong Z Ma G Zheng F Duan Y Ding S Luo A. Medication literacy in a cohort of Chinese patients discharged with essential hypertension. Front Public Health. (2019) 7:385. 10.3389/fpubh.2019.00385 31998676 PMC6962135

