# Supplementary material for: Status and influencing factors of medication literacy among Chinese caregivers of discharged children with Kawasaki disease
Source: Front Public Health. 2022 Oct 17;10:960913. doi: 10.3389/fpubh.2022.960913 (PMC9618952; doi:10.3389/fpubh.2022.960913)
Supplement: Supplementary file 1 [file Table_1.DOCX]

**Contents:**

1. **The relationship between KD patients and caregivers.**
2. **The flow chart of the survey process.**

**Appendix S1: The relationship between KD patients and caregivers.**

| **Role of the caregivers** | **Number of caregivers**  **No. (%)**  **(n=97)** |
| --- | --- |
| 1. Father | 17  (17.5%) |
| 1. Mother | 68  (70.1%) |
| 1. Grandfather | 2  (2.1%) |
| 1. Grandmother | 9  (9.3%) |
| 1. Others (auntie) | 1  (1.0%) |

**
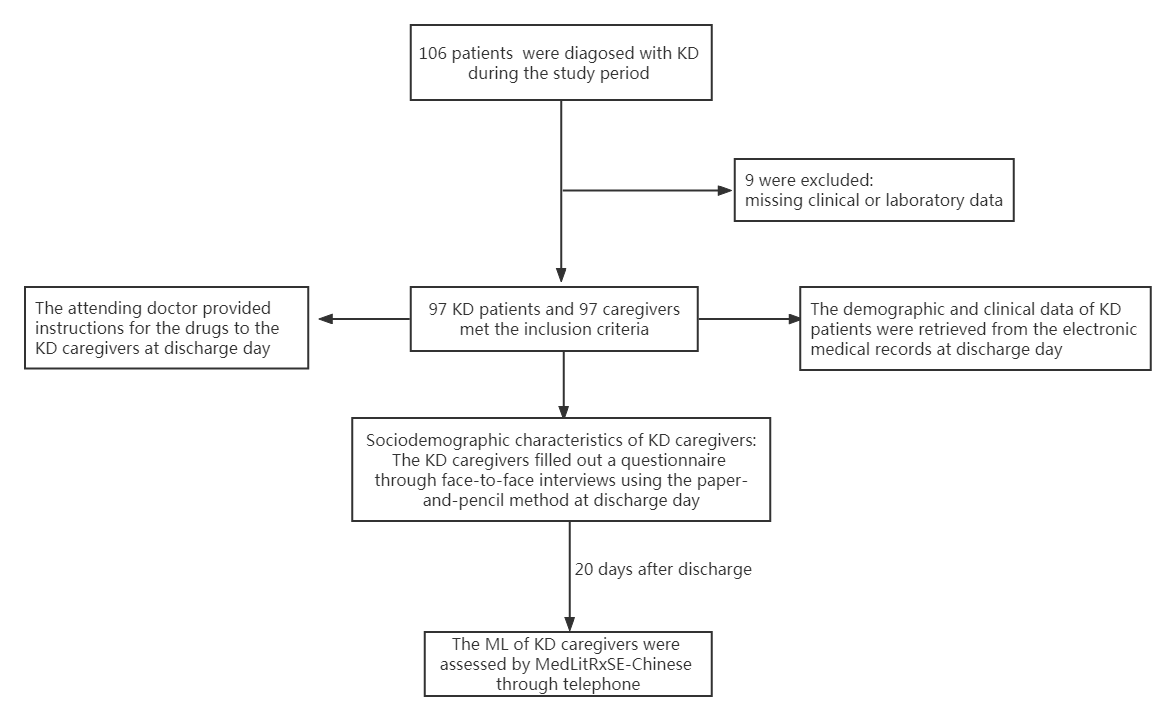
**

**Appendix S2: The flow chart of the survey process.**
